# 

**Published:** 1871-12

**Authors:** 


					﻿PUBLISHED MONTHLY, AT $3 PER ANNUM, IN ADVANCE.
.1	ATLANTA
5 I
OS I
I	hJ
.55	o
.Q	CD
I	8
1	Medical anil SurgicalJoiirnal J
.b	S
fc
*
s
<3^	§:
2	>1:
ft	EDITED BY	m
ft	p.
L
I	J. P. LOGAN, M.D.,
-3^
I	<:
5 W. K WESTMORELAKD, M.D.,
Ty_	'
S Professor of the Pnnc^les and Practice of Surgery in Atlanta Medical College, g
O	■ w
«o	o
S'
®	S 1
> 1
"	<Pax et Bcientia, sed veritas siive tim,ore.	S' i
s-------------------------------- §:
§	S'
1	VOLUilE LXB-DECEMBER, 1871.-NUMBER 9.	| i
S	CO
a	H
a	•	a.
o	?
o	®
i S	   p-
o	c*
(7*
£
<E
I	fJ'
p4	o
2	ATLANTA, GEORGIA:	?
g	PLANTATION PUBLISHING COMPANY.	” i
1871.	1
I
________EACH NUMBER CONTAINS SIXTY-FOUR PACES.	i
				

